# HTLV-1 Tax induces Th1 master regulator T-bet and thus IFN-γ in CD4+CCR4+ T-cells of virus-associated myelopathy patients

**DOI:** 10.1186/1742-4690-12-S1-P44

**Published:** 2015-08-28

**Authors:** Natsumi Araya, Tomoo Sato, Utano Tomaru, Ariella Coler-Reilly, Naoko Yagishita, Junji Yamauchi, Atsuhiko Hasegawa, Mari Kannagi, Hisanao Akiyama, Yasuhiro Hasegawa, Katsunori Takahashi, Yasuo Kunitomo, Yuetsu Tanaka, Atae Utsunomiya, Steven Jacobson, Yoshihisa Yamano

**Affiliations:** 1 Department of Rare Diseases Research, Institute of Medical Science, St. Marianna University School of Medicine, Kawasaki, Japan; 2 Department of Pathology, Hokkaido University Graduate School of Medicine, Sapporo, Japan; 3 Department of Immunotherapeutics, Tokyo Medical and Dental University, Graduate School, Tokyo, Japan; 4 Department of Neurology, St. Marianna University School of Medicine, Kawasaki, Japan; 5 Department of Immunology, Graduate School of Medicine, University of the Ryukyus, Okinawa, Japan; 6 Department of Hematology, Imamura Bun-in Hospital, Kagoshima, Japan; 7 Viral Immunology Section, Neuroimmunology Branch, National Institutes of Health, Bethesda, MD, USA

## 

The plasticity inherent to the CD4+ T cell differentiation program especially as it pertains to regulatory T (Treg) cells has been implicated in the pathogeneses of multiple inflammatory diseases. Human T-lymphotropic virus type 1 (HTLV-1) is thought to effect transcriptional changes in infected T cells via HTLV-1 Tax that can cause once suppressive CD4+CD25+CCR4+ Treg cells to lose FOXP3 expression and produce IFN-γ. We hypothesized that spawning of such inflammatory Th1-like cells from infected CCR4+ T cells plays a key role in the pathogenesis of the neurodegenerative inflammatory disease HTLV-1-associated myelopathy/tropical spastic paraparesis (HAM/TSP). In this study, we demonstrated that Tax in cooperation with specificity protein 1 (Sp1) boosts the expression of the Th1 master regulator T box transcription factor (T-bet/Tbx21) and consequently IFN-γ. We established the presence of abundant CD4+CCR4+ T cells co-expressing the Th1 marker CXCR3 and producing T-bet/Tbx21 and IFN-γ in the CSF and spinal cord lesions of HAM/TSP patients. Finally, we tested treatments on ex vivo cell cultures from patients and found evidence that a therapy targeting CCR4+ T cells via antibody-dependent cellular cytotoxicity may represent a viable treatment option for HAM/TSP.

